# Cryptic speciation and blurred species boundaries of the earthworm: A challenge for soil-based toxicological risk assessments

**DOI:** 10.1016/j.cbpc.2020.108880

**Published:** 2021-01

**Authors:** Andreas Katsiamides, Stephen R. Stürzenbaum

**Affiliations:** Department of Analytical, Environmental & Forensic Sciences, School of Population Health & Environmental Sciences, Faculty of Life Sciences & Medicine, King's College London, London, UK

**Keywords:** Earthworm, *Eisenia fetida*, *Dendrobaena veneta*, Cryptic speciation, OECD test

## Abstract

The toxicological risk assessment of chemicals is largely based on the Organization for Economic Co-operation and Development (OECD) guidelines. These internationally approved methodologies help shape policy and political strategy of environment and human health issues. Risk assessments which pertain to soil biota ‘recruit’ sentinel organisms, including the earthworm *Eisenia fetida*. Despite *E. fetida* being morphologically similar to *Dendrobaena veneta*, they are characterized by a several-fold difference in sensitivity to xenobiotics. Worms, sold as either as pure *E. fetida* stocks or *E. fetida*/*D. veneta* mixed cultures, were obtained from five commercial suppliers. The species identity of 25 earthworms was determined by sequencing the cytochrome *c* oxidase subunit 2 (COII). We revealed that only one of 25 worms was *E. fetida*, the remaining worms were all identified as *D. veneta*. This underlines the notion that *E. fetida* and *D. veneta* are easily mis-identified. The occurrence of cryptic speciation combined with the well-documented species-specific variation in toxicological responses highlights the pressing need to accurately classify earthworms to species level prior to any toxicological research. Only this will ensure the validity and reliability of risk assessments.

## Highlights

• The most frequently used worm in the standard OECD soil test is *Eisenia fetida*.
• The existence of a cryptic *E.fetida*/*andrei* species complex has previously been reported however *E.fetida* and *Dendrobaena veneta* are morphologically near identical.
• *E. fetida* is significantly more robust/resilient to chemical exposures than *D. veneta*.
• Toxicity tests based on the wrong species will provide misleading data.
• The risk of misidentification of earthworms may invalidate any OECD based tox tests.

## Graphical abstract

**Figure:**
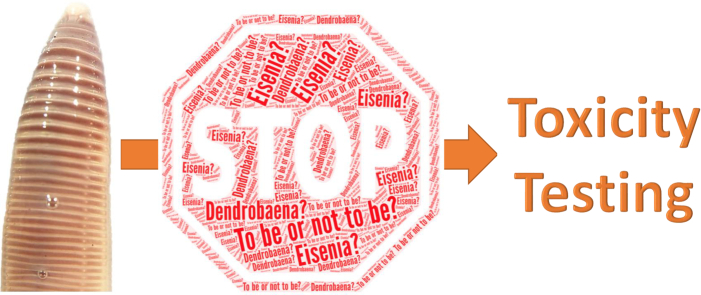

The use of experimental animals has undoubtedly helped shape our understanding of human disease but, unknown to many, they have also proven to be the cornerstone in the advancement of environmental risk assessments. The latter aims to assess to what extent drugs or agrochemicals impact on the aquatic and terrestrial ecosystem which in turn has direct consequences on mankind. National and international agencies, including the UK Environmental Agency, the United States Environmental Protection Agency (EPA) or the European Chemicals Agency (ECHA) have developed guidelines which are derived from standardized Organization for Economic Co-operation and Development (OECD) tests with the goal of assisting in shaping government policies and reforming political strategies around the environment and human health. The OECD Guidelines for the Testing of Chemicals is an extensive assembly of internationally approved methods which are routinely applied by independent laboratories to define risks and hazards of chemical exposure. Risk assessments pertinent to the soil biota typically involve either collembola (OECD 232) (Guimarães et al., 2019), mites (OECD 226) (Pierre et al., 2016), diptera (OECD 228) (Römbke et al., 2010), bees (OECD 213, 214, 237) (Dorigo et al., 2019), birds (OECD 205, 206, 223) (Edwards et al., 2017) plants (OECD 208, 227) (Lima et al., 2019) or earthworms (OECD 207, 220, 222) (Santos et al., 2017; Szabó-Fodor et al., 2017; Garcia-Velasco et al., 2016).

Earthworms are suitable indicators of soil health as they are exposed to chemicals externally (via dermal contact) and internally (via ingestion) but are also inherently involved in refining soil structure, water infiltration, and gas exchange, retention of nutrients, improvement of soil permeability and the reduction of erosion (Stürzenbaum et al., 2009). To our knowledge, all earthworm OECD tests specify the use of species resident to highly organic environments, typically *Eisenia andrei* or *Eisenia fetida*, with the vast majority of tests being performed on the latter. This aligns well with EGrowth, a global database collating information from 1073 growth curves encompassing some 51 earthworm species, which revealed that 22.7% of data was derived from the earthworm *E. fetida*, 8% from *E. andrei* and 2.1% from *Dendrobaena veneta* (Mathieu, 2018).

It is a well-established fact that toxicological profiles differ significantly between earthworm ecotypes inhabiting different parts of the soil column (Zhang et al., 2018) but this also applies to members of the same ecotype. For example, the mortality rate of the epigeic earthworms *E. fetida* and *Dendrobaena octaedra* challenged with the phosphorothioate insecticide Fenitrothion differ, with *E. fetida* being 8 times less sensitive to the compound than *D. octaedra* (Addison and Holmes, 1995). *E. fetida* is also less susceptible to exposures of glyphosate and the cholinesterase-inhibiting pesticide carbaryl than *D. veneta* (Jarmul-Pietraszczyk and Jastrzêbska, 2012). Likewise, *D. veneta* is, when compared to *E. fetida*, less resistant to exposure to heavily polluted sewage sludge (Suleiman et al., 2017). In contrast, bisphenol A *was shown to significantly impact* reproduction traits in *E. fetida but less so in D. veneta* (Verdú et al., 2018), a summary of examples is highlighted in Table 1. Observed differences in the response to toxic challenges are thought to be attributed to the distinct molecular genetic makeup that define the earthworm species (Stenersen et al., 1992; Fjøsne et al., 2015) or indeed due to species-specific microbial populations residing in the earthworm guts (Tica et al., 2013).

**Table 1:** Examples of species-specific sensitivities in earthworms exposed to toxic substances.

Addison and Holmes, 1995			Jarmul-Pietraszczyk and Jastrzêbska, 2012			Suleiman et al., 2017			Verdú et al., 2018		
Toxic substance tested	Species	LC50 (μg/ml)	Toxic substance tested	Species	LC50 (μg/dm^3^)	Toxic substance tested	Species	Bodyweight	Toxic substance tested	Species	Reproduction
Fenitrothion	*E. fetida*	393.9	Glifocyd 360 SL	*E. fetida*	320	Sewage sludge (including ~3 mg/kg Cd)	*E. fetida*	*D. veneta* exhibited a decrease in bodyweight vs an increase in *E. fetida*	Bisphenol A	*E. fetida*	A reduction in reproductive performance was observed in *E. fetida*
	*D. octaedra*	54.1		*D. veneta*	160		*D. veneta*			*D. veneta*	

Others have also highlighted the significance of cryptic speciation in invertebrates, due to differences in resistance to environmental stressors. For example, studies on the tubificid annelid, *Tubifex tubifex*, revealed five mitochondrial lineages, indicating the presence of morphologically indistinguishable cryptic species, each reported to have a distinct resistance to cadmium (Sturmbauer et al., 1999). Similar findings were reported concerning *Cletocamptus fourchensis* and *C. stimpsoni*, two cryptic species originally segregated from *C. deitersi*, which exhibit species-specific sensitivity to toxic heavy metals (Rocha-Olivares et al., 2004).

The marked species-specific variation in toxicological endpoints highlights the necessity to accurately classify earthworms to species level prior to any informative compound toxicology testing, especially if conducted as part of a legislative risk assessment. Morphological and phenotypic identifiers of earthworm species have been described (Sherlock, 2018), however only an experienced worm breeder or researcher will make an effort to differentiate between species. *Eisenia* and *Dendrobaena* are both characterized by red bands with yellow intersegmental spaces that lack dark pigmentation (Fig. 1A), thus superficially, they are morphologically indistinguishable and colour/pattern alone is a poor differentiator of species identity. However, the position of the setae, the hair-like bristles located on each segment, differ between the species, namely, they are more closely paired in *E. fetida* than in *D. veneta* (Fig. 1B and C). Scrutiny of setae position is a reliable means of species identification, but requires a microscope and is, at best, confirmatory within an assumed single species culture rather than an absolute identifier of worms within a complex multi-species culture. The definitive differentiation between species requires time-consuming classical mating experiments, the application of an electrophoretic approach to separate (acetyl)esterase patterns (Oien and Stenersen, 1984; Engelstad and Stenersen, 1991), or molecular barcoding (Pérez-Losada et al., 2005; Römbke et al., 2016). Some have argued that even 16S and COI based sequence analyses are only able to discern taxonomic differences above the genus level and call for combinatory approach that also encompass morphological, biological, physiological, ecological indices. Most studies conducted to date have focused on the need for (and associated challenges of) tools that allow the simple and reliable differentiation of earthworm within the *E. fetida*/*E. andrei* species complex, in particular due to the fact that *E. fetida* is one of the primary invertebrates chosen for OECD tests.

**Fig. 1:**
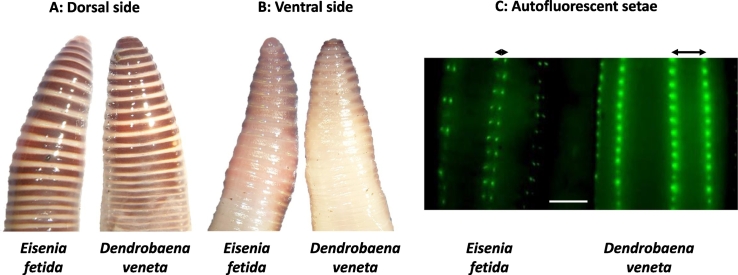
Phenotypic characterization of *Eisenia fetida* and *Dendrobaena veneta*. A. The dorsal sides demonstrates the phenotypic similarity, both species being striped with interchanging layers of brown and yellow. B. Setae are positioned on the ventral side where the pigmentation is greatly diminished. C. The setae can be readily visualized by utilizing their intrinsic capacity to autofluoresce (images were digitally captured using a Nikon microscope SMZ800 with blue laser scanning fluorescence (lex = 450–490 nm)). The arrows indicate the distance of the setae within each pair; Note the setae are closely paired in *E. fetida* and widely paired in *D. veneta*. Scale bar 200 μm.

This current study builds on the notion that species identification is of paramount importance and that phenotypic analysis alone can be misleading, not limited to the *E. fetida*/*E. andrei* species complex, but also beyond. We determined the species identity of earthworms obtained from five commercial suppliers in the United Kingdom marketing the sale of a pure *E. fetida* culture (companies A and B) or mixed culture consisting of *E. fetida* and *D. veneta* (companies C to E). From each supplier, we randomly selected five worms and anaesthetized them by immersion in cold carbonated water. Pharyngeal tissue was isolated by dissection and total RNA extracted using the Quick-RNA™ MiniPrep kit (Zymo Research, Irvine, USA). Following the synthesis of cDNA, the cytochrome *c* oxidase subunit 2 (COII) was amplified by PCR (sense: 5′-CAAGATGCCGCATCTTCTG-3′; anti-sense: 5′-ACGGCATCTACTTTTACGCC3′). The resulting amplicons were Sanger sequenced (Genewiz, UK), submitted to the NCBI database (Genbank accession numbers MN552401–MN552425), aligned by ClustalW and a phylogenetic tree built using the Maximum Composite Likelihood (MCL) method and the Tamura-Nei algorithm (MEGAX), including COII sequences of *E. fetida*, *E. andrei*, *Lumbricus rubellus*, *L. terrestris* and *D. veneta* from global locations (obtained either from NCBI or specimens from collaborators) to account for potential sequence variations due to geographical distance.

Of the 25 worms sourced from the five UK companies, only one worm (from company A) was a genuine *Eisenia* species, the remaining 24 were grouped within the *Dendrobaena* clade (Fig. 2). Nine of the ten worms sampled from companies marketing their stocks as pure *Eisenia* cultures (companies A and B) were *Dendrobaena* species and all worms sampled from companies claiming to supply *Eisenia* within a mixed culture were identified as *Dendrobaena*. The phylogenetic tree also revealed that *L. rubellus* and *L. terrestris* are closer to *Eisenia* species than *Dendrobaena* species, despite *Eisenia* and *Dendrobaena* being morphologically more similar (*Lumbricus* does not display the contrasting banding pattern but is characterized by a uniform pigmentation). Furthermore, there was some overlap between the *E. fetida* and *E. andrei* sequences, suggesting that the two species are either isogenic or that some published sequence data is based on the incorrect species identification.

**Fig. 2:**
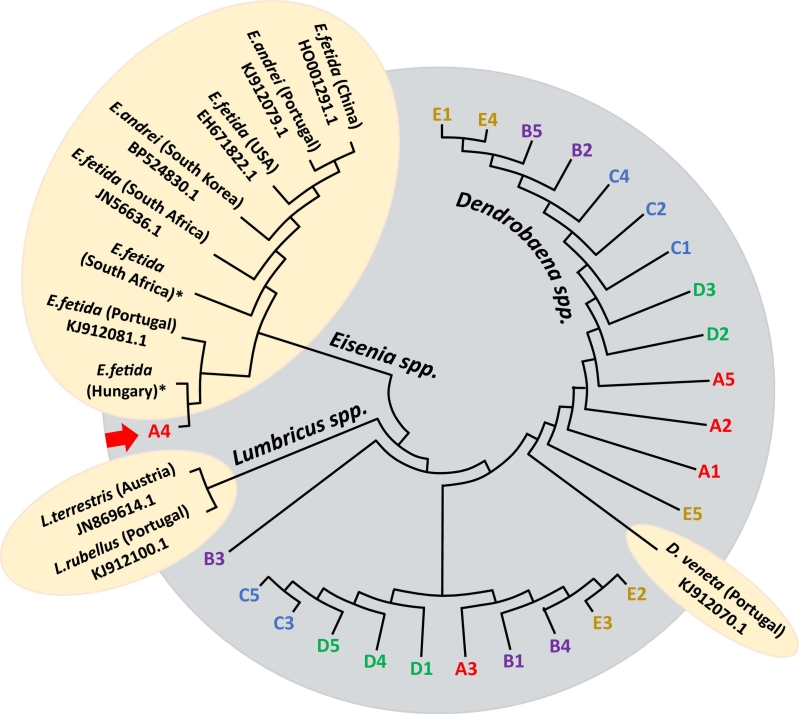
Phylogenetic classification of earthworms based on their cytochrome *c* oxidase (COII) sequence. Randomly selected individuals sourced from five UK suppliers claiming to sell either pure *Eisenia fetida* (companies A and B) or a mixture of *E. fetida* and *Dendrobaena veneta* (companies C to E) were obtained and the cytochrome *c* oxidase gene sequenced (Genbank accession numbers MN552401–MN552425), then compared to published reference sequences from NCBI (https://www.ncbi.nlm.nih.gov). The evolutionary relatedness was inferred by using the Maximum Likelihood method and Tamura-Nei model in MEGAX. *E.fetida obtained from collaborators in South Africa and Hungary, respectively.

Species mis-identification is by no means a novel finding and has been reported by others. Römbke et al., 2016, for example, conducted a comprehensive barcoding analysis of *E. fetida* and *E. andrei* stocks across 28 ecotoxicological test laboratories and in doing so revealed that only 61% of worms were correctly identified. The findings from this present paper highlight that cryptic speciation is not limited to the *E. fetida*/*andrei* complex but applies equally to *D. veneta*. Namely, most worms provided by UK suppliers are *D. veneta* and not, as claimed, *E. fetida*. The significance and impact of this observation is by no means trivial as species mis-identification, combined with the documented species-specific differences in biological responses can have significant implications for toxicological research, namely either significantly under- or over-estimating the toxic profile of xenobiotics (Römbke et al., 2016).

We therefore reiterate the pressing need to identify earthworms to species level prior to any (eco)toxicological testing. Regular barcoding of stocks to re-confirm species composition of stocks may be time-consuming but essential check point. Arguably too ambitious at this stage, but highly desirable, would be the establishment of a centralized ‘worm banks’, derived from fully characterized founder populations, as is available for biomedical model organisms (e.g. Bristol (N2) is classified as the *C. elegans* wild-type strain). An equivalent earthworm stock would be ideally suited to act as a “wild-type” reference, thereby removing a major confounding variable originating from overlooked cryptic speciation. In short, this paper aims to remind the scientific community and risk assessors that comprehensive species identification is a fundamental necessity prior to any experimental use of earthworms. Only then will OECD tests be truly predictive, robust, reproduceable and valid.

## Funding

This work was funded by the BBSRC London Interdisciplinary Doctoral Programme (LIDo) [grant number BB/M009513/1].

## Declaration of competing interest

The authors declare that they have no known competing financial interests or personal relationships that could have appeared to influence the work reported in this paper.
